# Large extensional earthquakes push-up terrific amount of fluids

**DOI:** 10.1038/s41598-022-18688-6

**Published:** 2022-08-26

**Authors:** Claudio Chiarabba, Pasquale De Gori, Luisa Valoroso, Marco Petitta, Eugenio Carminati

**Affiliations:** 1 INGV, Istituto Nazionale di Geofisica e Vulcanologia, Rome, Italy; 2 Dipartimento di Scienze della Terra, Sapienza University of Rome, Rome, Italy

**Keywords:** Geophysics, Seismology, Tectonics

## Abstract

How large earthquakes are triggered is a key question in Earth science, and the role played by fluid pressure seems to be crucial. Nevertheless, evaluation of involved fluid volumes is seldom investigated, if not unaccounted for. Moreover, fluid flow along fault zones is a driving factor for seismicity migration, episodic heat and chemical transport. Here we show that time repeated (4D) seismic tomography resolves changes of V_p_ and V_p_/V_s_ during the Mw6.2 2009 L’Aquila normal faulting sequence, that indicate a post-failure fluid migration from hypocentral depths to the surface, with a volume estimated between 5 and 100 × 10^6^ m^3^ rising at rates up to 100 m/day. This amount inferred by tomograms is surprisingly consistent with the about 50 × 10^6^ m^3^ surplus water volume additionally measured at spring discharge, spread in time and space along the 700 km^2^-wide regional carbonate fractured aquifer. Fluids were pushed-up within a huge volume across the fault and expelled from the area of large coseismic slip. Such quantities of fluids liberated during earthquakes add unprecedented constraints to the discussion on the role of fluids during and possibly before earthquake, as well as to the potential impact on the pristine high-quality drinkable groundwater, possibly affecting the biodiversity of groundwater dependent ecosystems too.

## Introduction

Fluid chemistry and flux rates are sensitive to the seismic cycle and are particularly variable during coseismic and postseismic stages^1–9^. Fluid pressure variation along fault zones is a potential mechanism for earthquake triggering^2^, episodic heat and chemical transport^4^, modulating large shocks^3,10^ and seismicity migration^11^, also associated with geo-energy activities^12^.

Although the role of fluids is largely complimented in the literature, direct observations are rare, and the mechanism of earthquake-fluid interaction is still unclear. Over-pressurized fluids at hypocentral depth are inferred by P and S-wave velocity anomalies and faulting mechanisms^6,13^. Velocity changes identified by time lapse tomography are used to infer cracks opening during strong ground shaking^14^, changes of material properties and seismicity migration along fault systems^15^, fluid diffusion processes before or after major ruptures^16,17^. Further evidence comes from in situ observations^4^, water-level changes in wells^18^, variations of fluid flow in springs and wells^19^, geochemical variations of water^20–22^ satellite radar interferograms^18^. Finally, evidence of paleo fluid flow induced by seismic cycles comes from studies of mineralizations from exhumed fault zones^23^.

Even if all these observations agree on the primary role of fluids in the seismogenic process, quantification of fluid flow associated with earthquakes is practically unknown, limiting a full understanding of the mechanism and strength of fluid-rock interaction during seismic cycles. The assessment of fluid volumes involved in the earthquake process is crucial to foster future studies on precursory signals of earthquakes^9^ and evaluate the potential impact on drinkable water pollution^7^ and biodiversity^8^.

**Figure 1:**
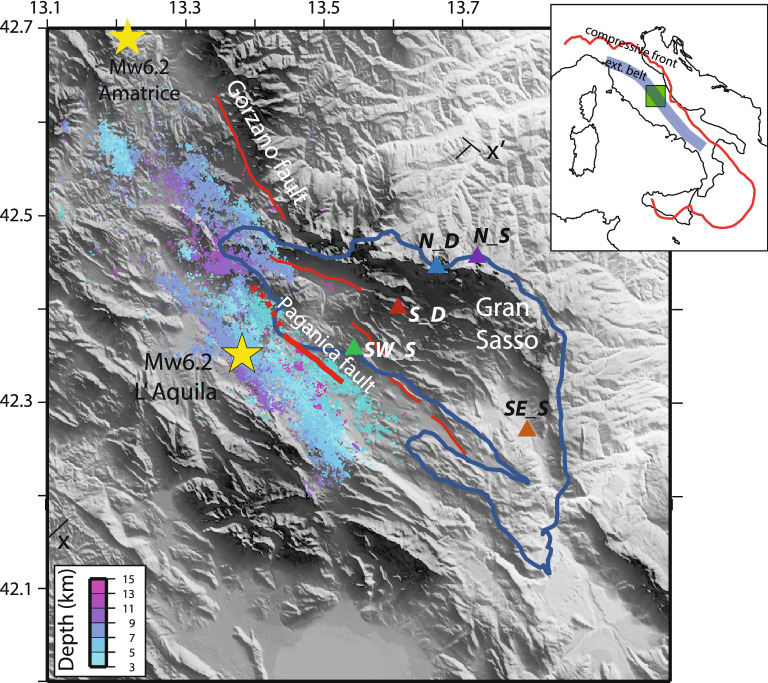
Map showing the topography of the area, earthquake epicenters from April to December 2009^27^, the lateral limit of the Gran Sasso aquifer (blue line) and the location of the analyzed springs. The yellow stars are the Mw6.2 2009 L’Aquila and Mw6.2 2016 Amatrice epicenters. The endpoints of sections in Fig. 2 are shown. Red lines are the main normal faults, in bold the Paganica fault activated during the 2009 mainshock. Figure has been done with Generic Mapping Tool software, https://www.generic-mapping-tools.org/.

In this study, we integrate high quality seismological^5^ and hydrogeological^24,25^ data from the L’Aquila 2009 Mw6.2 earthquake (Fig. 1) to quantify, for the first time, the fluid diffusion process after a large earthquake. The fault is located at the boundary of a wide fractured and karstified regional aquifer, with large seasonal and interannual phases of groundwater recharge/discharge^26^.

The 2009 L’Aquila normal faulting event shows a strong rupture complexity^28^, intense post-seismic slip and fault segment interaction^27,29^. Notable changes in fluid flow at hypocentral depth prior to the earthquake, indicated by V_p_/V_s_ changes during the foreshock sequence, were proposed to play a key role in earthquake nucleation^30,31^. Co-seismic and post-seismic changes in groundwater discharge and hydrochemistry were inferred from springs analyses^25^, showing long-lasting increase in discharge in some springs and a general spreading of hydrogeochemical and chemical-physical (e.g., pH, electrical conductivity, calcite saturation index) anomalies migrating with time from the epicentral area to the entire hydrogeological basin.

In this study, we map the variation of material properties on the L’Aquila fault and surrounding volumes for a period of two months after the 2009 mainshock. We adopt a time-lapse tomographic approach that computes deviations from a static 3D model, as described in the Method section. The high-quality dataset has been subdivided into three sub-epochs (Fig. S1, Table S1 of the Supplementary Material), following variations observed in the trend of P and S-wave residuals after location in a 3D static model (Fig. S2). We then discuss the variations of V_p_ and V_p_/V_s_ in terms of post-seismic fluid diffusion along the fault system, following predictions by faulting models^32,33^. The upwelling velocity and amount of fluids mobilized from depths is then quantified and compared with hydrogeological information resulting from water budget.

## Results

The static 3D model presents strong anomalies both in V_p_ and V_p_/V_s_ (Fig. S3). A sharp contrast in V_p_/V_s_ is observed across the fault at the depth of the mainshock, with high V_p_/V_s_ in the carbonate volume in the footwall of the fault (Fig. S4). From this static model, we observe that the temporal changes of anomalies in the three epochs are significant (4–5%), but still a small fraction of the lateral changes in the static model. Therefore, the gross structure and fluid state and content is given by the static model, while the relatively small changes observed during the three epochs account for time changes of physical parameters or fluid content and pressure.

**Figure 2:**
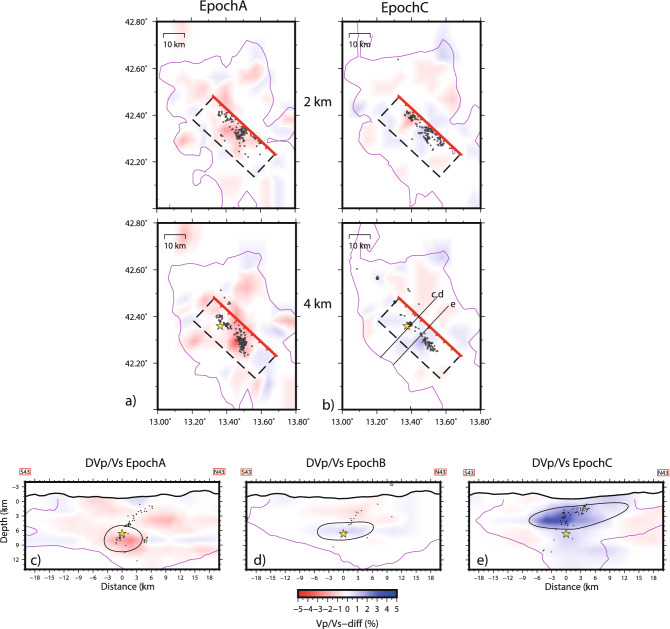
Map of V_p_/V_s_ at 2 and 4 km depth for EPOCHA (**a**) and EPOCHC (**b**). Purple lines are the limit of the resolved volume. The box of the fault (plotted in Fig. 4) is shown, with epicenters at + /− 1 km from the layer and the mainshock epicenter. The traces of the vertical sections are indicated. Vertical sections across the 2009 fault of V_p_/V_s_ for the three distinct epochs (**c**–**e**). Section e in EPOCHC is drawn 4 km south of sections c and d to capture the most significant time change of V_p_/V_s_, since the migration occurred both along strike and upward. Aftershocks identify the fault geometry at depth. The well resolved volume is indicated by the thin purple curves.The zones with pronounced anomalies are encircled, showing a first V_p_/V_s_ decrease and a progressive increase of V_p_/V_s_ at shallower depths. Figure has been done with Generic Mapping Tool software, https://www.generic-mapping-tools.org/.

Figure 2 shows the difference of V_p_/V_s_ anomalies for the different epochs in map (for the more significant layers) and vertical sections. The complete V_p_ and V_p_/V_s_ suite of models for the three epochs are shown in Figs. S6, S7, and S8 of the Supplementary material. The most significant feature is the change of V_p_/V_s_ at hypocentral depths (2–10 km) during the three epochs. Sharp variations are visible both along the fault plane and in the enclosing volume, and V_p_/V_s_ reverses from low to high between EPOCHA to EPOCHC (Fig. 2a, b). A progressive increase of V_p_/V_s_ up to 5% is observed between EPOCHA and EPOCHC around and on the fault (Fig. 2a, b), in a well-resolved volume of the model. A first V_p_/V_s_ decrease during EPOCHA (Fig. 2c), reversed in a V_p_/V_s_ increase during EPOCHB and EPOCHC (Fig. 2d, e) is visible close to the nucleation of the 2009 rupture and in the volume around it. The progressive shallowing of the high V_p_/V_s_ anomaly in the hanging-wall of the mainshock fault is also evident (Fig. 2d, e).

### Hydrogeological budget

**Figure 3:**
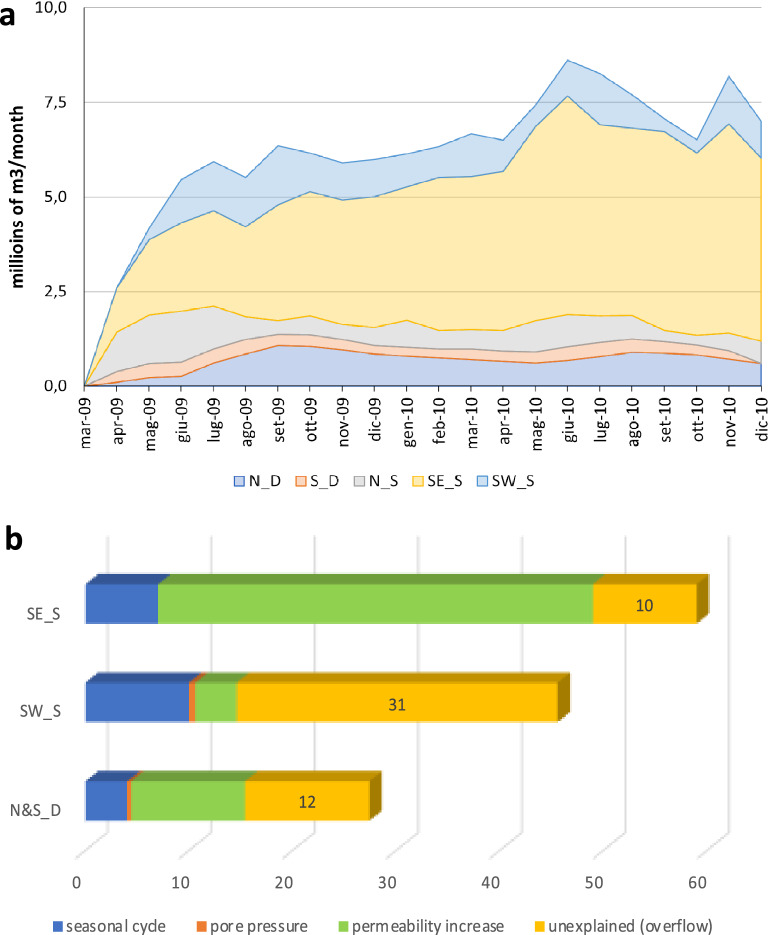
Spring IDs refer to Fig. 1. (**a**) (upper panel): Post-seismic monthly discharge increase of the monitored springs (location in Fig. 1). The surplus discharge has been calculated from the starting time of March 2009. The values represent the discharge increase of each month with respect to the previous month.The other springs of the Gran Sasso aquifer have recorded only limited changes (increase and decrease) in the weeks following the mainshock, without long-lasting effects. (**b**) (lower panel): Decomposition of the surplus in spring discharge (in millions of m^3^) for selected springs, triggered by the April 6th, 2009 earthquake, with identification of the overflow discharge as equivalent to the deep fluid rising contribution to discharge of selected spring (yellow bars).

The Gran Sasso aquifer^24–26^ feeds about 15 main springs (mean discharge ranging between 0.1 and 5 m^3^/s) located at its boundaries, with minor springflow on the northern side (0.1–0.7 m^3^/s) and larger base flow in the southwestern (0.2–0.9 m^3^/s) and mainly in the southeastern areas (2–5 m^3^/s). An additional groundwater drainage corresponds to the highway tunnels crossing the core aquifer, having about 1.5 m^3^/s of discharge, equally distributed on both sides. A significant long-lasting increase of the spring discharge has been observed mainly on the southern side (SW_S and SE_S in Fig. 1) and along the tunnel drainage (S_D and N_D in Fig. 1), with respect to changes recorded on the northern springs, limitedly affected by the post-seismic changes (e.g., N_S in Fig. 1). The comparison between pre-mainshock and historical discharges (with a long-term average of 600 × 10^6^ m^3^ per year) and those recorded since April 2009 to December 2010 (about 20 months), highlights a total increase in discharge of about 130 × 10^6^ m^3^, a huge surplus water volume sourcing from the Gran Sasso aquifer after the mainshock. The discharge increase has been calculated on a monthly basis, considering March 2009 as initial reference and comparing the post-seismic recharge of the main springs. Monthly volume with respect to the previous month rapidly grows in the first three months and then it continues to constantly flow until December 2010, as shown in Fig. 3a.

To distinguish the different contributions to the total discharge, a multiple hydrograph separation has been realized for each spring group. We distinguished four contributions: (a) the natural baseflow due to the normal regime of the spring^24^, (b) the sharp co-seismic increase due to the pore-pressure propagation in the aquifer immediately after the mainshock; (c) the additional drainage from the aquifer to the springs due to a rise in bulk hydraulic conductivity determined by the cleaning effect on fractures and related flow by the pore-pressure propagation, as known in literature^34^; (d) a residual increase in discharge not attributable to the mentioned processes, which can be considered equivalent to the deep source of fluids injected in the aquifer (“deep overflow”).

The baseflow of each hydrograph is represented by the natural groundwater cycle, and the related discharge is due to the infiltration and recharge processes on the aquifer outcrops. This component has been calculated from pre-mainshock long-term mean discharge^24^, with the addition of a seasonal effect typical of each spring, calculated by the interval between minimum and maximum values along the 2009–2010 years.

The aliquot attributed to the sharp co-seismic increase in the fractured aquifer is clearly visible in each hydrograph, also in other not shown springs, and it has a duration no longer than two months. The effect of the increased bulk hydraulic conductivity was not directly calculated but assumed from existing studied: we adopted an increase of 16% in hydraulic conductivity, in agreement with an estimation of 10–20% provided by 24, which has been refined after the Amatrice-Norcia earthquake in 2016^35^, assumed to be congruent for the L’Aquila earthquake, due to the similar hydrogeological context and similar magnitude of the mainshock.

By difference of the post-mainshock measured discharge with the above-mentioned aliquots, the residual unaccounted increase in spring discharge from April 2009 to December 2010 has been finally evaluated. The length of the considered period was limited to 20 months after the mainshock, because spring discharge at the end of 2010 went back to values like the pre-seismic period, except for SE_S where longer effects have been observed for the following years and attributed to the bulk hydraulic conductivity effect.

Consequently, we attribute the total surplus to the following different processes (Fig. 3b): the seasonal cycle of the spring discharge accounts for about 20 × 10^6^ m^3^; a rough estimation of the pore pressure effect corresponds to a negligible discharge release, not higher than 1 × 10^6^ m^3^; the effect of pore-pressure propagation and consequent hydraulic conductivity transient increase would explain about 60 × 10^6^ m^3^, mainly long-lasting recorded at the base flow drainage system SE_S; the remaining surplus of about 50 × 10^6^ m^3^ is attributed to “deep overflow” and is compared in the following with the volume of migrating fluids inferred from tomography.

## Discussion

Hydrological changes following major normal fault earthquakes have been discussed for some cases worldwide Muir-wood^36^. We contribute to this topic by quantifying the volume of fluids mobilized after a large normal faulting earthquake, thanks to a joint analysis of seismological and hydrogeological data.

**Figure 4:**
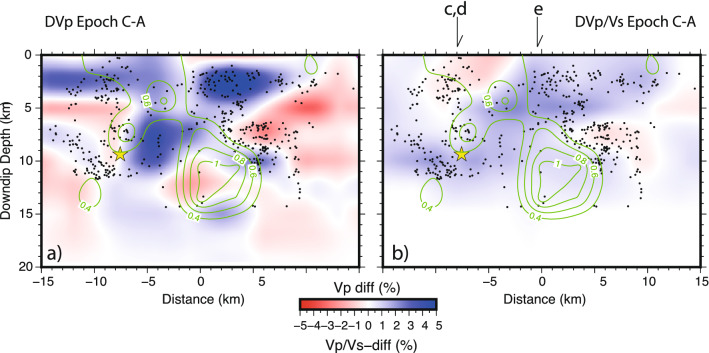
Velocity changes, V_p_ (**a**) and V_p_/V_s_ (**b**), between EPOCHC and EPOCHA along the fault. The star is the mainshocks, black dots are aftershocks occurring at + /− 0.5 km from the fault. Green lines indicate the coseismic slip countours (in m) of the 2009 main shock. The patches with high coseismic slip correspond to V_p_ decrease, suggesting the dehydration of the fault plane. A general increase of V_p_/V_s_ on the fault plane, mostly in its upper portion, is observed. Figure has been done with Generic Mapping Tool software, https://www.generic-mapping-tools.org/.

Time-resolved transient changes of V_p_/V_s_ observed after the L’Aquila earthquake are strong and might be interpreted as sharp variations of pore fluid pressure induced by the earthquake^10^. The V_p_/V_s_ decrease at hypocentral depth is followed and balanced by a reciprocal increase in the surrounding volume suggesting fluid discharge from the volume around the fault for the intense cracking induced by slip and their migration within the high V_p_ carbonate body (Figs. 2 and 4). Fault decarbonation might also account for additional circulation of CO_2_ enriched fluids^34,37^.

Di Stefano et al.^38^ showed that the mainshock nucleated within a negative poisson ratio patch with an insignificant coseismic slip. A relative increase in EPOCHB, soon after the mainshock, corresponds to a progressive uprise of fluids (V_p_/V_s_ increase) from a source zone below the high V_p_, low V_p_/V_s_ volume of the carbonatic succession. We propose that fault slip induced notable fluid migration toward the surface^32,33^ highlighted by velocity changes during the aftershock sequence. Most likely these fluids were accumulated at hypocentral depth during the earthquake preparatory phase^31^. The dimension of the volume involved in the process indicates that fluid flow is not only channeled by enhanced fracturing and secondary permeability within the fault damage zone, that is about 0.5 km wide^27^, but it rather occurs in a wider, up to 10 km, rock volume. Excluding also linear poroelastic effects, inconsistent with the amount of fluid discharge^9^, permanent deformation within a large volume is to be invoked. Consistent with 3D numerical models for the l’Aquila earthquake^39^, we suggest that fault slip may have caused volumetric contraction in a 6–8 km-wide volume on the fault asperity, indicated by a V_p_ reduction, and fracture-assisted dilation (V_p_/V_s_ increase) at shallower levels (Fig. 4). Expulsion of fluids from hypocentral depths and migration to the surface are caused by this permanent deformation, associated with the rupture of a seal topping the over-pressured fluid compartment. Fluid migration was accommodated by progressive cracking, as indicated by the fact that most of the aftershocks^29^ occurred in volumes with highest V_p_/V_s_ (and fluid) increase. This process is consistent with the time change of shear wave velocity observed during the sequence by ambient noise tomography^40^.

The surplus water volume reaching the springs (Fig. 3) from deep sources was calculated at about 50 × 10^6^ m^3^, with an expected predominance at SW_S (30 × 10^6^ m^3^), located close to the seismogenic fault. Much less expected, but consistent with the large extent of V_p_/V_s_ anomaly, N&S_D (highway tunnel drainages that, due to their anthropic nature, can intercept groundwater flow from any direction) received about 10 × 10^6^ m^3^ of the deep overflow. Springs on the northern side (N_S), far from the deep overflow origin, only show limited changes in discharge, and they were affected mainly by a pore-pressure propagation effect on short term. In SE_S, the natural destination of the base-flow of the whole Gran Sasso Aquifer, the increase from deep overflow was slightly delayed, smoother, and longer-lasting, owing to the time necessary for the fluid wave to move there from the seismogenic fault area.

A rough estimate of the upwelling deep fluids volume can be derived from tomographic images. The ca. 250 km^2^ wide high V_p_/V_s_ anomaly imaged by tomography (see the positive V_p_/V_s_ anomaly in EPOCHC in layers of Fig. 2) must be extended at least 10 km along the fault strike. Therefore, the positive V_p_/V_s_ time change progressively migrating and expanding toward the surface involves a rock volume of at least 2500 km^3^. Values of the specific storage of saturated rocks between 2 and 5 km depth have been limitedly evaluated in past research: literature estimation ranges between mean values of 2 × 10^–6^ m^−1^ with maximum values of 4 × 10^–5^ m^−1^^41^). These mean values agree with upper values indicated for fault zones in carbonate aquifers^42^, ranging from 2 × 10^–8^ to 1.6 × 10^–6^ m^-1^. From these estimates, the expected water/fluid volume contained in the upwelling velocity anomaly volume ranges between 5 and 100 × 10^6^ m^3^, and the average of this evaluation corresponds to the measured 50 × 10^6^ m^3^ deep overflow obtained from hydrogeological surplus data.

According to the shift of the positive V_p_/V_s_ time change (Fig. 2c–e), deep fluids are raised with very fast velocities (reaching about 100 m/d between EPOCHA and EPOCHB). This velocity is faster than the maximum groundwater velocity (35 m/d) recorded for fast flow components along main faults of the aquifer in interseismic periods^26^. This velocity difference is explained by the combination of different seismically-enhanced mechanisms (volume contraction at depth and expansion at shallower depths, increase of permeability along the faults), but it is probably aided by additional factors such as dissolved gas carrying capacity, like CO_2_. Once at shallow depth (< 1 km), the natural increase in the horizontal permeability^35^ contributed to spreading the fluids in a wide volume (EPOCHC).

In this framework model, some considerations are relevant:

- fast rising fluids moved along the fault during co-seismic period, but they involved a much larger volume in post-seismic period, with dispersion in the aquifer volume, not only limited along the fault direction;
- the post-seismic uplift of the deep overflow has a long-lasting (still relevant after 20 months) effect on aquifer discharge, decreasing in volume with time;
- the estimated overflow volume is of the same order of magnitude as that recorded for the Amatrice 2016 seismic sequence (around 400 × 10^6^ m^3^^43^).

### Pro and cons of fluid flow: precursory signatures and potential water pollution

The increase of the permeability moving towards the surface is supposed to be a major driver of the observed spreading of the V_p_/V_s_ anomalies with time. Aquifers in fractured and partially karstified carbonates show hydraulic conductivity some orders of magnitude higher in the first 1–2 km than at 2–10 km depths^35^. With raising, the dispersion of deep fluids and related flow paths into shallow rocks is likely enhanced, due to the expected permeability increase, up to four orders of magnitude; this results in the involvement of a larger volume of the hit aquifer. This process can have effects on groundwater quality:

1. Uprising fluids have chemico-physical characteristics able to enhance the water–rock interaction (e.g. lowering pH), possibly leaching metal ions along the flowpath towards surface; this can lead to an increase of their concentration in groundwater also before the mainshock, as recorded for the Amatrice 2016 earthquake^22,42^ at springs located at the southern boundary of the Gran Sasso aquifer and even up to 100 km far from the epicenter (Popoli Gorge^22^). In this case, Arsenic, representative of heavy metals anomalies, increased its concentration over the drinking water threshold, up to 20 times the natural pre-earthquake content for a period of 9 months, and a total concentration of 2 kg/L (Fig. S13). Based on such analogue, we infer a potential release of some tons of Arsenic in the Gran Sasso groundwater during and after the L’Aquila earthquake. Even if we do not expect a similar Arsenic content in the whole aquifer, assuming a limited spread of the same Arsenic mass on the 10% of the mean discharge of the aquifer (based on the spring discharge at the station closer to the fault: 1.8 m^3^/s), an amount of 3600 kg of Arsenic was potentially released from the deep fluids. The concentration in Arsenic of the deep fluids able to contaminate the springs could be estimated in about 70 microg/L. In this scenario, type of ion enrichment depends on fluids characteristics and bedrock composition, and consequently an increase in specific ions can be useful to trace back the source with depth. Similar anomalies were measured before the Amatrice 2016 seismic sequence and, although observed in springs as far as 100 km from the hypocenter, were potential precursory signatures^22^. Although our study is referred to post-seismic flow, the described spreading of deep fluids flux at shallow depth over distances of tens of km across the fault is consistent and helps to explain why seismically induced geochemical anomalies can be found also at large distance^22^ from the hypocenter.
2. In terms of groundwater quality, every change due to deep fluids spreading into the aquifer, can consistently affect the chemical content of groundwater resources frequently used for drinking purposes^7^; even though for transient and limited periods, the increase of metal undesired ions (e.g., Cd and As) can hinder the drinking use representing an additional side effect of post-seismic conditions to be taken into account, as recorded after the Amatrice 2016 seismic sequence in springs located very close to the Gran Sasso aquifers. Changes of groundwater geochemistry may also drive to loss of biodiversity^8^, as suggested by the dramatic decrease in subterranean Copepoda species abundance recorded in the Presciano spring (SE_S, Fig. 1), ascribable to pore-pressure propagation along fractures and microkarst channels and /or changes in chemical-physical parameters.

## Conclusions

Seismic and hydrogeological data show that a terrific volume of fluids (around 50 × 10^6^ m^3^) was pushed up toward the surface following the Mw6.2 2019 L’Aquila earthquake. We hypothesize that time changes of velocity anomaly might indicate local pore pressure variations associated with this flow, possibly responsible for the occurrence of large aftershocks (M_L_ > 4) and postseismic features. We conclude that about 50 × 10^6^ m^3^ of fluids (mainly groundwater) were progressively mobilized at least from depths of ca. 5 km up to the surface, feeding the groundwater cycle for about 20 months following the mainshock.

Fluid diffusion processes play a crucial role in the spatiotemporal evolution of seismic sequences and the volume of mobilized fluids, attempted here for the first time, helps elucidating the mechanism of fluid-earthquakes interaction with impact on potential precursory signatures and groundwater quality, having potential negative effects both on drinking water supply and on biodiversity.

To sum up, these findings are useful both to:

- advance our knowledge of the triggering mechanism process in co- and post-seismic periods, not forgetting the possibility to take advantage of pre-seismic fluid migration as potential signatures for incoming earthquake.
- consider among post-seismic emergencies, the need to check changes in groundwater quality and quantity, which can hinder drinking use and can have negative effects on the biosphere.

## Methods

### Seismic data and methods

Tomographic models have been computed by using P- and S-waves arrival times, recorded by a dense local network of 108 permanent and temporary stations, from all aftershocks with M_L_ > 1.9 occurred throughout 2009 after the April 6th Mw6.2 L’Aquila mainshock (details on the starting aftershocks catalog are described in^5^). From this dataset, we selected 2059 events having location errors less than 1 km and azimuthal gap < 180°. P-wave and S-P arrival times were then inverted using the Simulps14 algorithm^44^. Part of this dataset (i.e., the first three months of the aftershocks sequence) includes the events used to retrieve a 3D velocity image of the 2009 L’Aquila fault^35^.

Crucial for time-lapse tomography is the a priori definition of time windows and epochs to subdivide the dataset. Different strategies are used and discussed in the literature. We adopt here a subdivision into non- overlapping consecutive periods, with a windowing that is based on the analysis of trends in arrival times residuals but keeping as much as possible the similarity in number of data. For the time lapse tomography, we have followed the approach in which the entire dataset is first inverted for obtaining a static 3D model with the aim to include all the stationary spatial perturbations^45^. The 3D static model is plotted in Figs. S3, S4 and S5 in map and vertical sections. Then, we compute residuals of P and S-P arrivals through the 3D velocity model to verify the existence of transient signals (i.e., part of the data that does not fit in the 3D model, see Fig. S2). The trend in P and S-P residuals shows distinct and peculiar features that we used for framing the epoch subdivision. We observe that the aftershocks occurring in the first days (days 6 to 24) present clear transient V_p_/V_s_ signals. On the contrary, the oscillation around null values, expectable for a random data unfitting, is only visible in the last part of the sequence (after day 24; Fig. S2). This behavior clearly reflects the fact that the static 3D model does not fit adequately the data during the first days, probably because of transient velocity changes superimposing on the 3D features.

Therefore, we subdivided the dataset into three sub-epochs: EPOCHA (April 6–13) contains the largest positive residuals and V_p_/V_s_ variation; EPOCHB (April 13–24) still shows significant positive and negative residuals variation and V_p_/V_s_ variations while during EPOCHC (April 24–May 31) we observe small oscillation around the mean 3D model. The inversion of the three epochs is done using the static 3D model as the input model of the inversion and using similar damping values. In this way, the velocity changes modeled in each epoch are the only necessary to better fit the data, while artifacts, eventually very small in our case, due to ray path different covering are minimized using the common 3D starting model. Earthquakes used in the three inversions have a similar spatial distribution (Fig. S1) and sample as homogeneously as possible the volume. This aspect can be critical since hypocentral differences between earthquakes of different epochs may lead to sample crustal volumes with different properties, resulting in apparent temporal changes of velocity. In our case, the time lapse approach used and the good ray sampling in the central part of the model contribute to avoiding this artifact.

Statistical data for the three inversions are reported in Table S1. The final rms and variance improvement obtained by the three inversions is similar. Thus, the velocity models obtained using the same inversion setting although showing significant differences fit equivalently the inverted data (in each epoch). The location rms for all the earthquakes is smaller than those achieved using a single model to fit all the observations, indicating that the sub-epoch models match the data more closely.

The closer the resolution between the different epochs, the higher is the reliability of space–time anomaly changes. Although an identical volume sampling is hardly obtainable by using local earthquakes, a time-consistent resolution is today a viable way to verify the reliability of results. In this study, we have computed the full resolution matrix^46^, selecting the same value of spread function (which encompasses the well resolved volumes) as the limit for comparing results, and performed synthetic tests for both V_p_ and V_p_/V_s_ models (see Supplementary material). The full Resolution matrix for all the three epochs have been computed and analysed. By visually inspecting the averaging vectors, plotting DWS (derivative weight sum) vs the spread function^46^ and mapping the contour of the 70% of the diagonal elements^46^, we select a very conservative value of the spread function threshold for well resolved parameters (SF = 1.5) much smaller than the value indicative of good resolution by the analysis (SF = 2.0 see Fig. S9). In all the tomographic images of the different epochs, we plot the SF = 1.5 line and discuss variation of anomalies only within the similarly resolved volumes.

To further assess the consistency of model resolution between the three epochs, we have also computed synthetic tests that simulate:

- **Checkerboard tests for V**_**p**_** and V**_**p**_**/V**_**s**_ (Fig. S10);
- **A synthetic V**_**p**_**/V**_**s**_** anomalies resembling the final model** at 8 km depth (i.e., Restore test, Fig. S11).

Tests have been done adding a random noise equal to the final standard deviation of the real inversion and using similar damping parameters of the real inversions.

All tests show that the synthetic V_p_ and V_p_/V_s_ anomalies are similarly reproduced, both in geometry and amplitude, within the three epochs. Difference in amplitude of the recovery anomalies between the three epochs is less than 5%. Results from the resolution matrix and synthetic tests similarly indicate that the resolution is good within the central region of the model and consistent among the three different epochs. We are therefore very confident on the reliability of space–time anomaly changes.

### Hydrogeological data

Data from spring discharges related to the 2008–2010 period (Fig. S12) have been collected by several sources. Daily data of the highway Gran Sasso tunnel drainages (both the north and the south tunnels, N_D and S_D respectively) and of Vacelliera (Ruzzo) spring group (N_S) arrive from Drinking Water Authorities^25^. The discharges of Tempera and Vetoio groups (SW_S) have been measured at monthly scale before and after the mainshock^25^. The Tirino River discharge (SE_S), corresponding to the bigger group including Capodacqua and Presciano springs, have been evaluated by the official river gauging station “Tirino a Madonnina”, located in Bussi sul Tirino village^24^. The total mean discharge of selected springs is about 9 m3/s, representing half of the total groundwater discharge of the Gran Sasso aquifer (18 m3/s). Discharge from other springs were not available with the same detail, but many of the not-considered springs, where data are available, show only a short-term discharge increase, due to the pore-pressure propagation, with a negligible volume increase after the mainshock, also due to their limited mean discharge. Consequently, we consider representative of the post-mainshock anomalies in discharge the above selected springs (as shown in Fig. 1).

The discharges recorded before and after the mainshock, summarized in^25^, clearly demonstrated that post-mainshock discharge is higher than pre-mainshock values, and this additional amount in percentage is decreasing with distance from the causative fault. The increase for the closest springs (e.g. SW_S, S_D) ranges between 30 and 45%, while the other considered springs limit their increase from 8 to 16%.

## Supplementary Information

Supplementary Information.

## Data Availability

Waveform data can be retrieved on the EIDA seismic database, http://eida.ingv.it/it/getdata. Earthquakes data and velocity models are accessible at ftp.ingv.it/pub/pasquale.degori/COMMSENV-21-0561-T.

## Code availability

The seismic tomography software code is free and a version can be downloaded at http://faldersons.net/Software/Simulps.
