# Supplementary material for: Large extensional earthquakes push-up terrific amount of fluids
Source: Sci Rep. 2022 Aug 26;12:14597. doi: 10.1038/s41598-022-18688-6 (PMC9418187; doi:10.1038/s41598-022-18688-6)
Supplement: Supplementary file 1 — Supplementary Information. [file 41598_2022_18688_MOESM1_ESM.pdf]

Supplementary Material

Large extensional earthquakes push-up terrific amount of fluids

Chiarabba Claudio[1], De Gori Pasquale[1], Valoroso Luisa[1], Petitta Marco[2], Carminati Eugenio[2]

[1]INGV, Istituto Nazionale di Geofisica e Vulcanologia, Rome, Italy

[2]Dipartimento di Scienze della Terra, Sapienza University of Rome, Italy

Contents of this file

Table SOM1

Figures SOM1 to SOM13

Table SOM1

Inversions	n. eq.	n. P	n. S	Variance improvement	Final rms
EPOCHA 6-13/4	734	17050	8803	18,00%	0.16 s
EPOCHB 13-24/4	557	18395	9462	22,00%	0.12 s
EPOCHC 24/4 - 31/5	768	24347	11940	27,00%	0.11 s

Table SOM1 Statistical data for the three inversions: n. eq.: number of earthquakes; n. P: number of P-wave arrivals; n. S: number of S-wave arrivals.

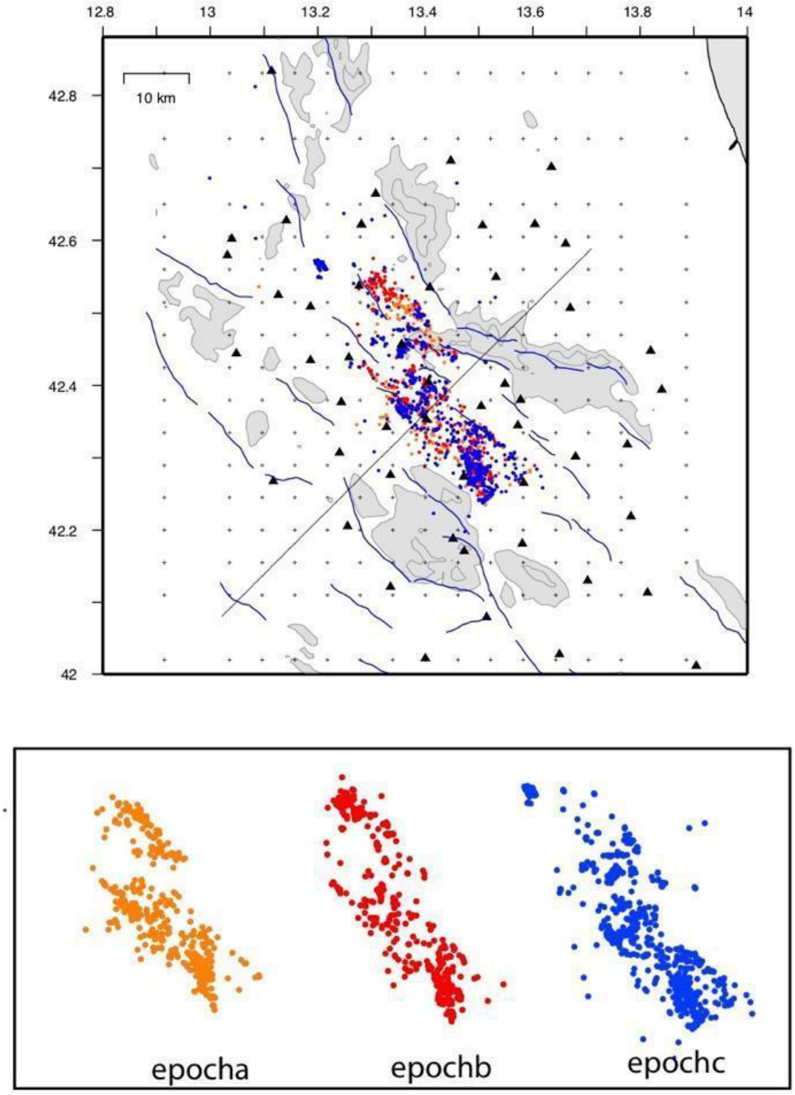

Figure SOM1: Top panel) Seismic stations (triangles), model grid nodes (crosses) and earthquakes (dots color coded based on the three epochs) used in the inversion. The cross section shown in the main text is plotted. Gray masks are elevation higher than 2000 mt. Lower panel) Zoom on the earthquake distribution for the three epochs

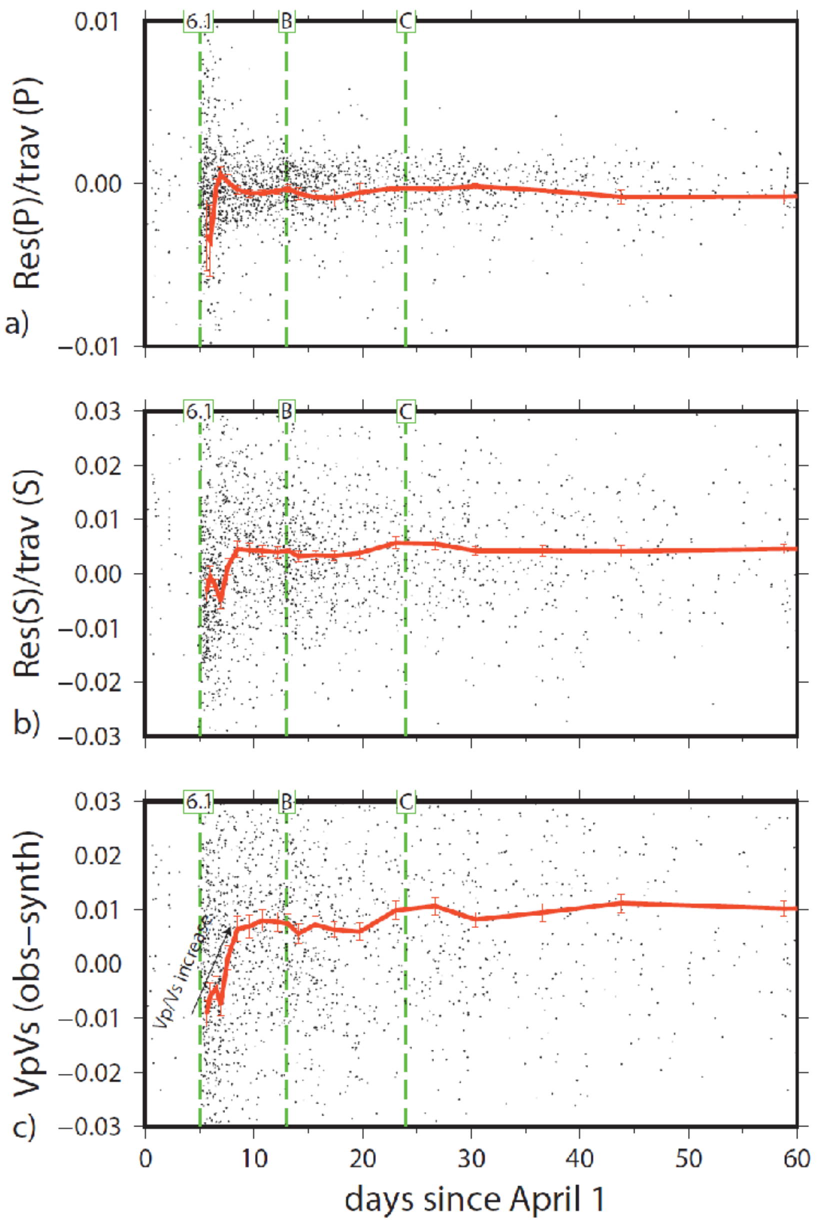

Figure SOM2: P- (a), S- (b) and Vp/Vs residuals (c) of events vs time (since April 1 2009), after the location with the 3D model. The mean P- and S-wave residuals of each event, normalized by the traveltime to account for different ray path-lengths, are smoothed by a moving average of 200 points (events) shifted forward by 100 points. Standard deviation is shown. Note the strong transient signals in the first part of the sequence. Based on such trends we subdivided the dataset into the three epochs (indicated here with green bars).

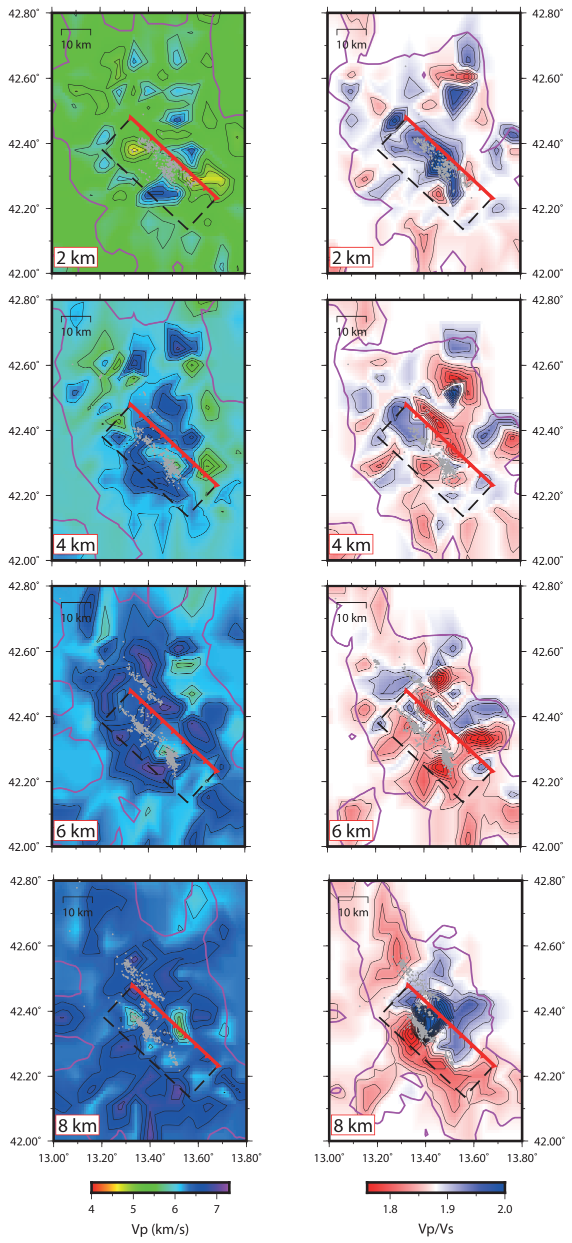

Figure SOM3: The 3D static Vp and Vp/Vs models in layers from 2 to 8 km depth. Aftershocks are plotted in gray dots, the fault projection at the surface is plotted. The limit of the well resolved areas (SF=1.5) is shown by the purple line.

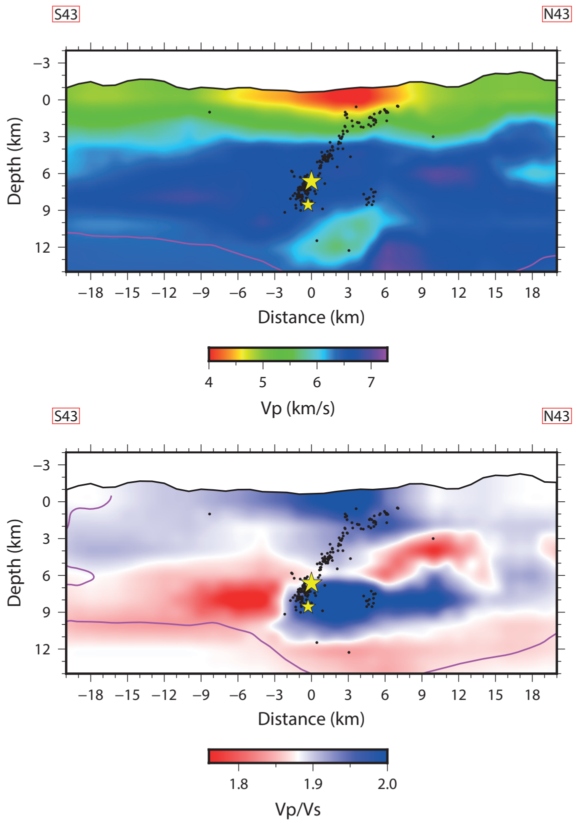

Figure SOM4: Vertical section of Vp and Vp/Vs of the 3D static model at the mainshock hypocenter. Aftershocks are plotted in black dots, the yellow star is the mainshock location, the smaller star a M4 foreshock. The limit of the well resolved areas is shown by the purple line (SF=1.5).

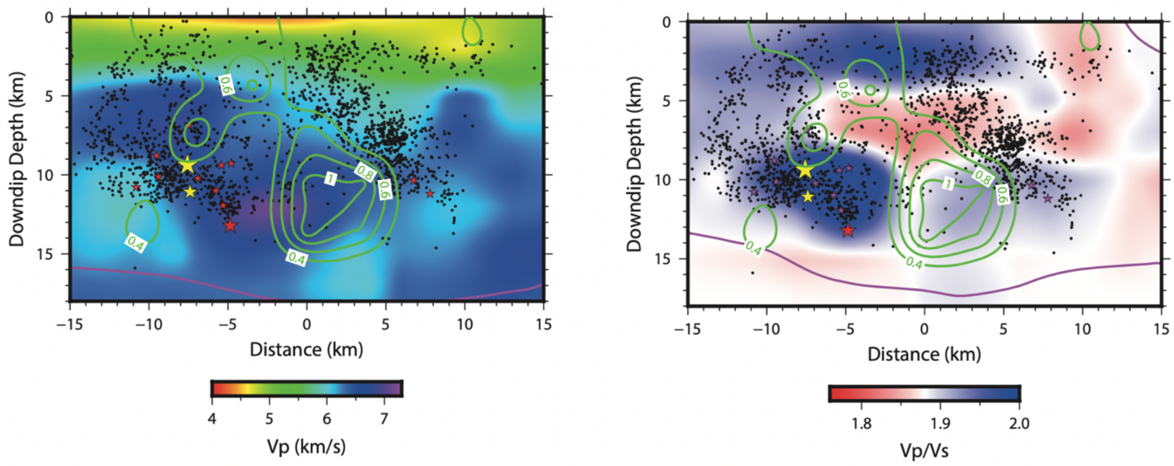

Figure SOM5: Downdip section of the 3D static Vp and Vp/Vs models along the fault. M4 foreshock (small yellow star), mainshock (yellow star) M>4 aftershocks (red stars) M<4 aftershocks (black dots), and coseismic slip (in meters) from Cirella et al. (2012) are shown. The limit of the well resolved areas is shown by the purple line (SF=1.5).

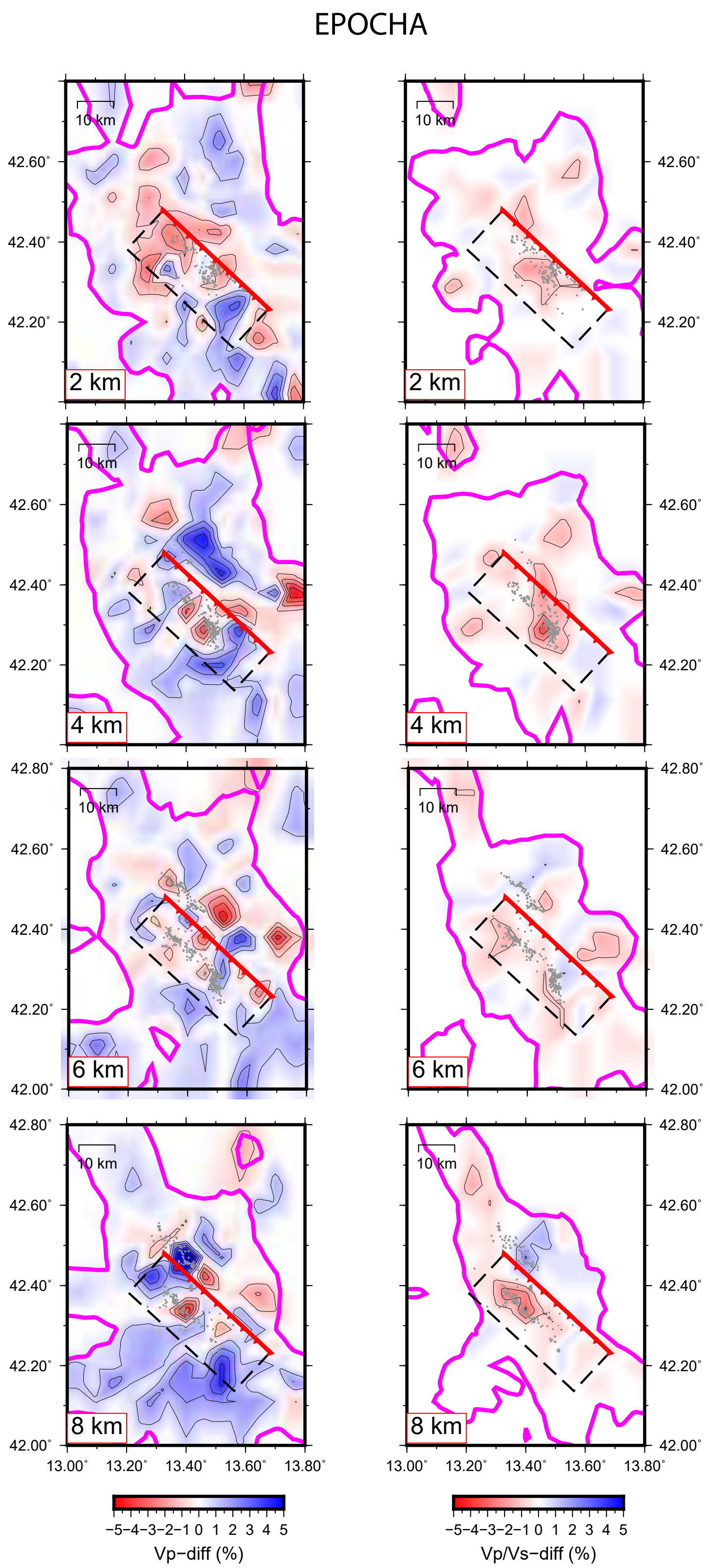

Figure SOM6: Difference in Vp and Vp/Vs between EPOCHA and the static 3D model for layers between 2 and 8 km depth. Purple line is the limit of the resolved region (SF=1.5). Grey dots are earthquakes that occurred in the epoch. The fault box is indicated.

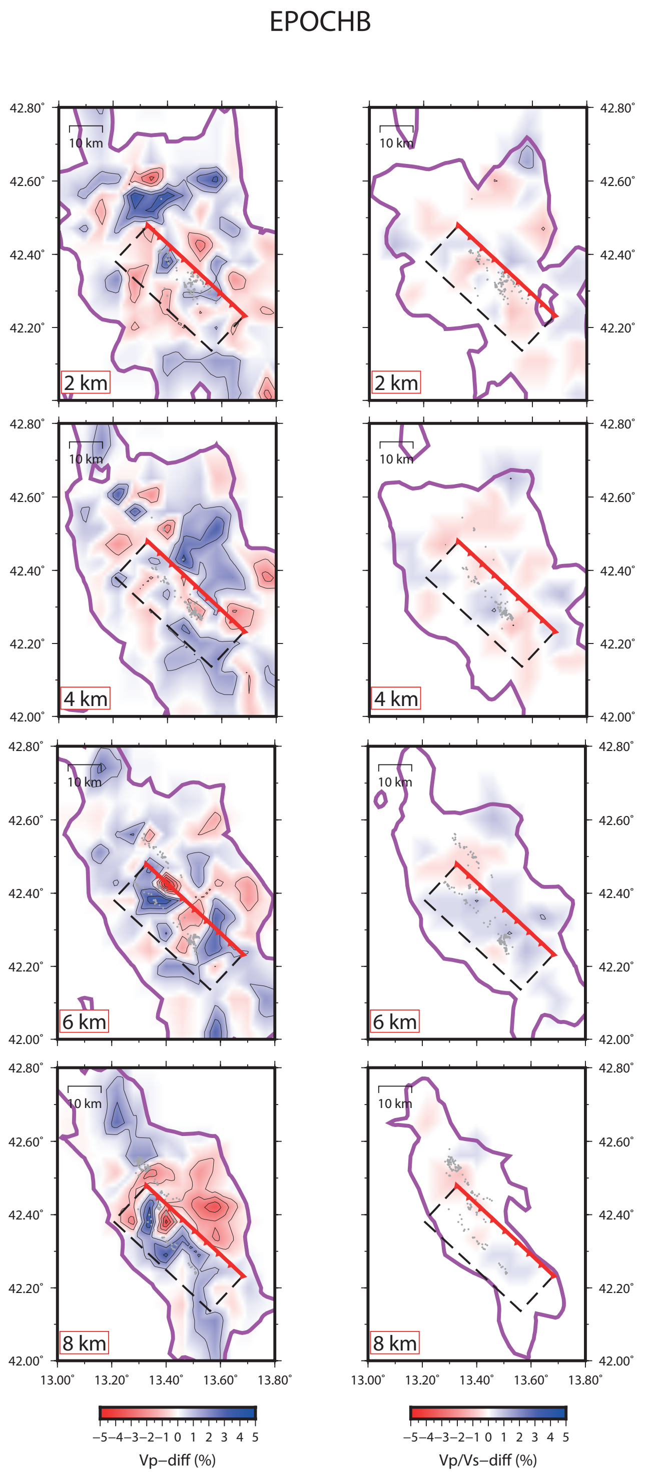

Figure SOM7: Difference in Vp and Vp/Vs between EPOCHB and the static 3D model for layers between 2 and 8 km depth. Purple line is the limit of the resolved region (SF=1.5). Grey dots are earthquakes that occurred in the epoch. The fault box is indicated.

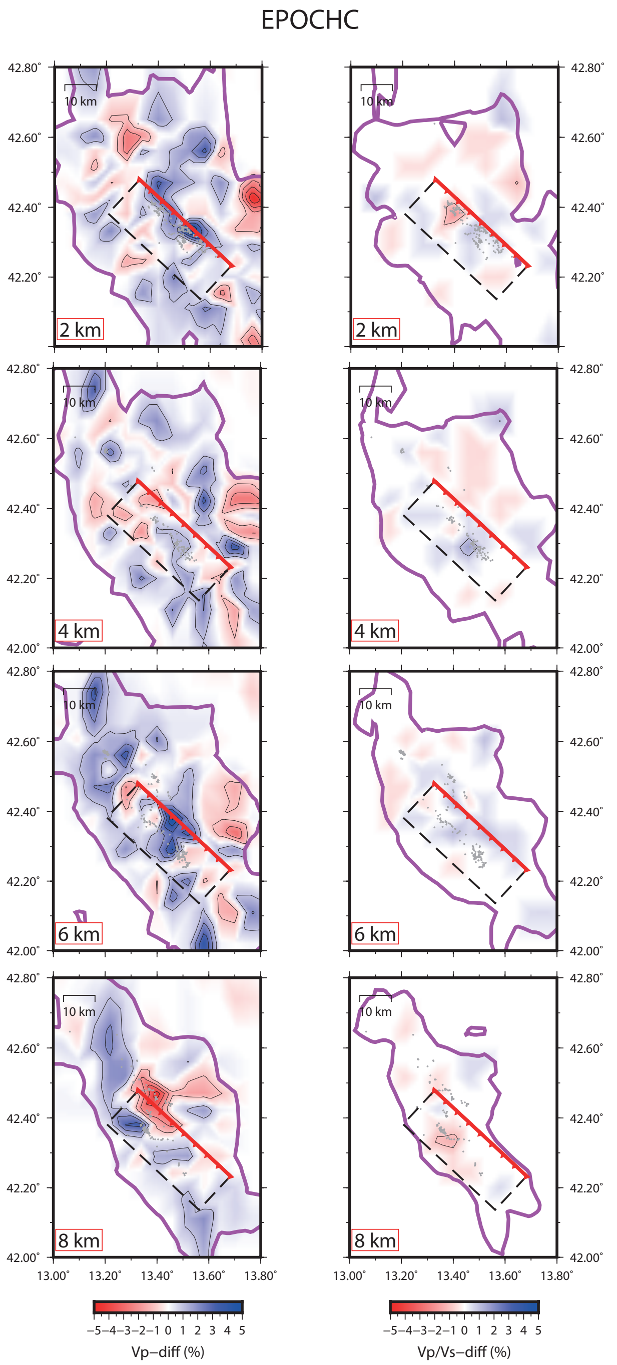

Figure SOM8: Difference in Vp and Vp/Vs between EPOCHC and the static 3D model for layers between 2 and 8 km depth. Purple line is the limit of the resolved region (SF=1.5). Grey dots are earthquakes that occurred in the epoch. The fault box is indicated.

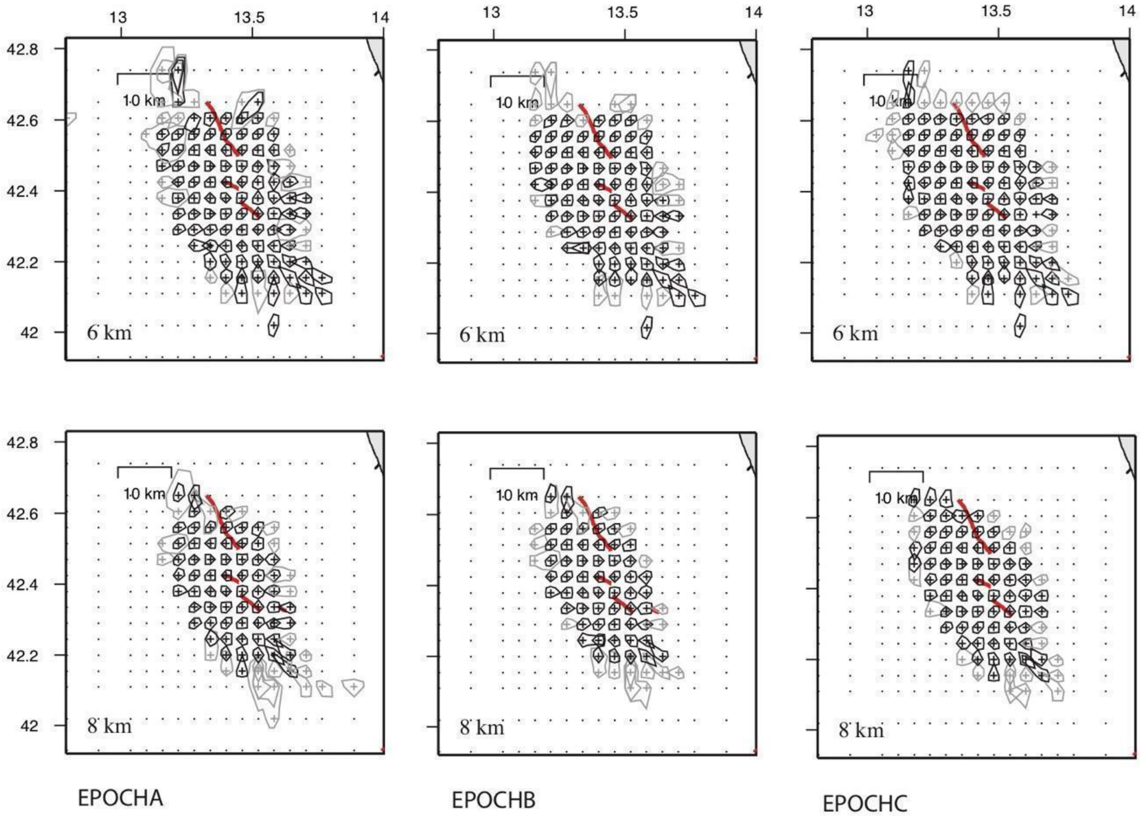

Figure SOM9: Map of the 70% volume for each velocity node of the Vp model at 6 and 8 km depth for the three different epochs. Black lines denote well resolved nodes. Main faults (Paganica and Gorzano) are shown by the red lines.

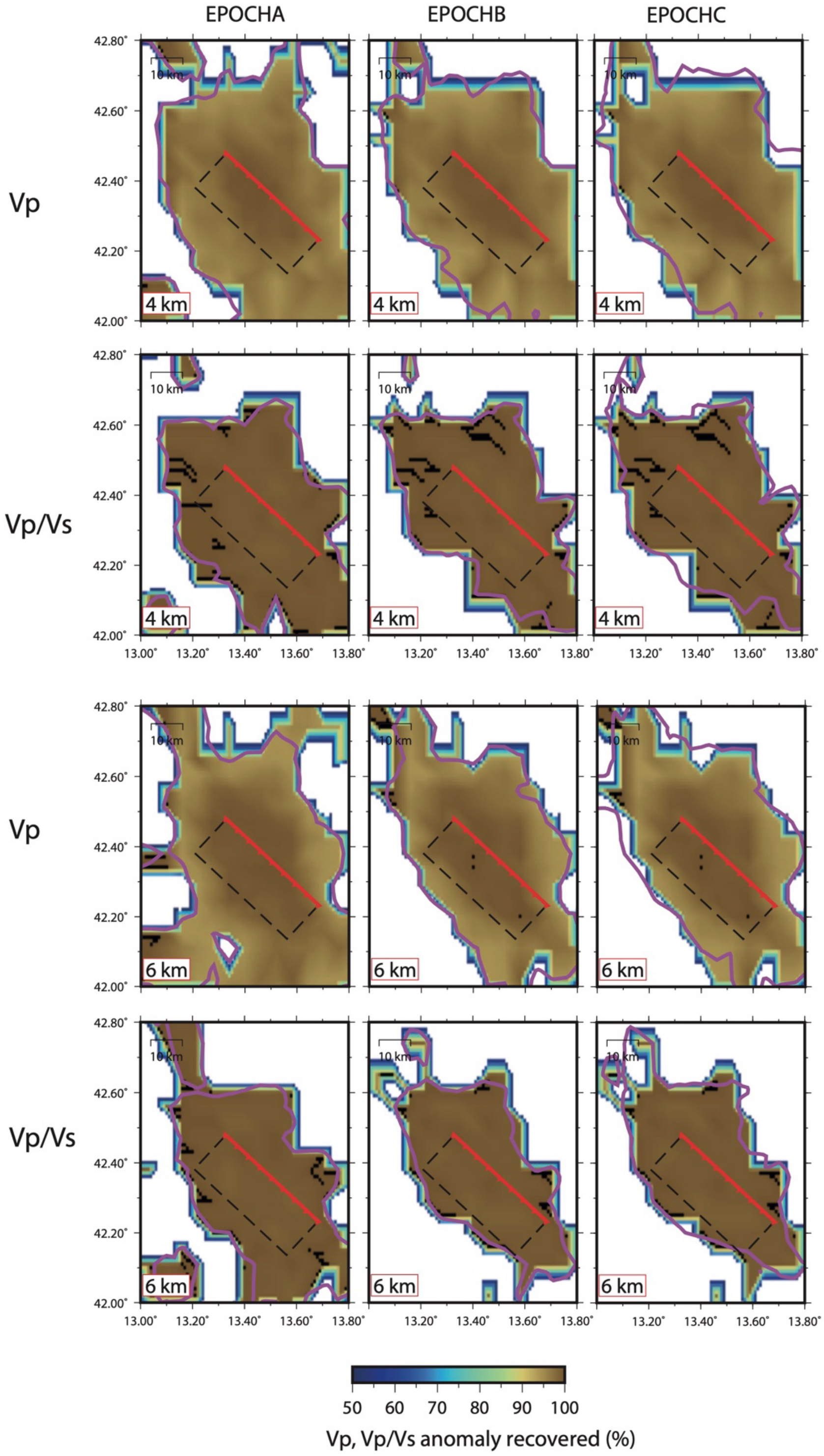

Figure SOM10: Synthetic test showing the percentage of recovery of synthetic features for Vp and Vp/Vs at 4 and 6 km depth for the three epochs. The box indicates the L'Aquila fault. The purple line is the SF1.5 isoline.

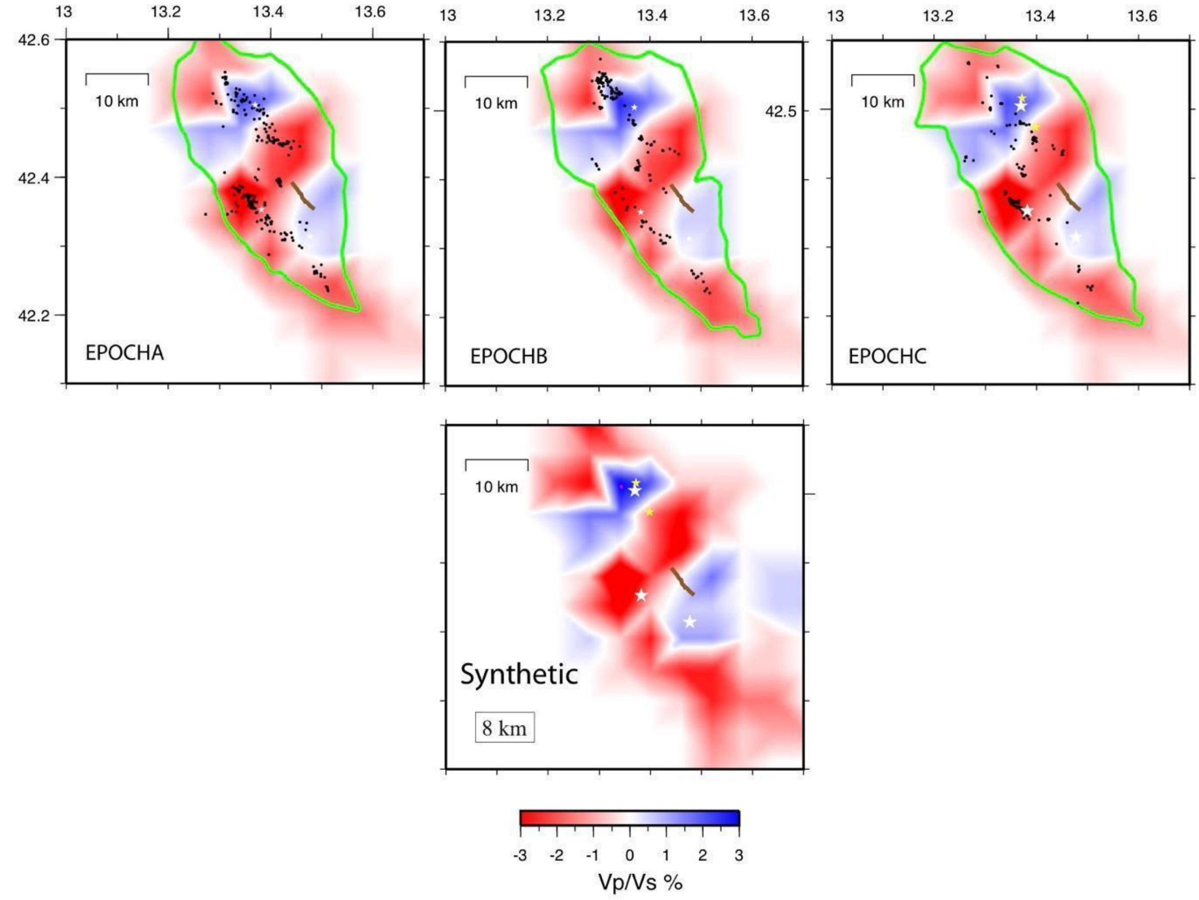

Figure SOM11: Synthetic test simulating synthetic Vp/Vs anomalies (bottom panel) at 8 km depth. The green line is the SF1.5 isoline, the brown line is the Paganica fault.

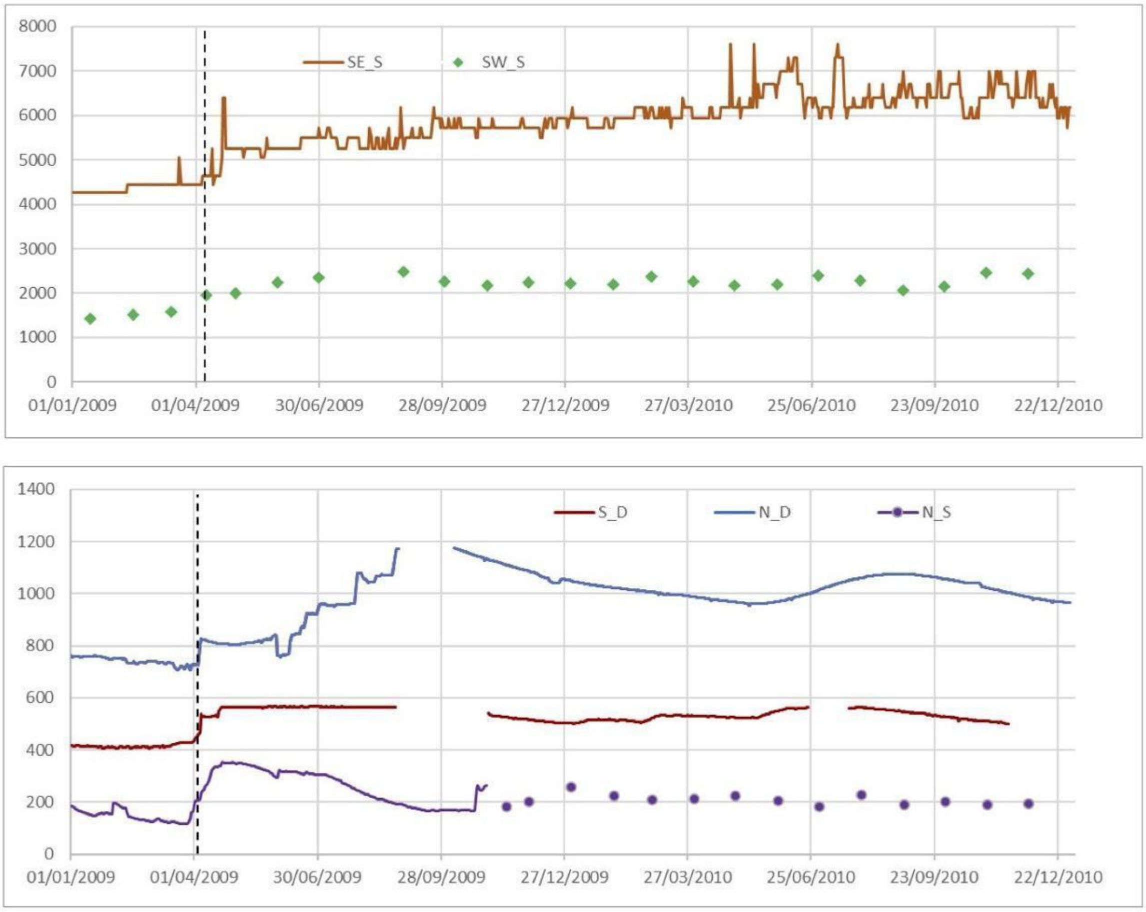

Figure SOM12: 2009-2010 discharge (in L/s) of selected springs. Coloured lines identify daily data availability, while dots correspond to monthly data. Dotted black line represents the earthquake date.

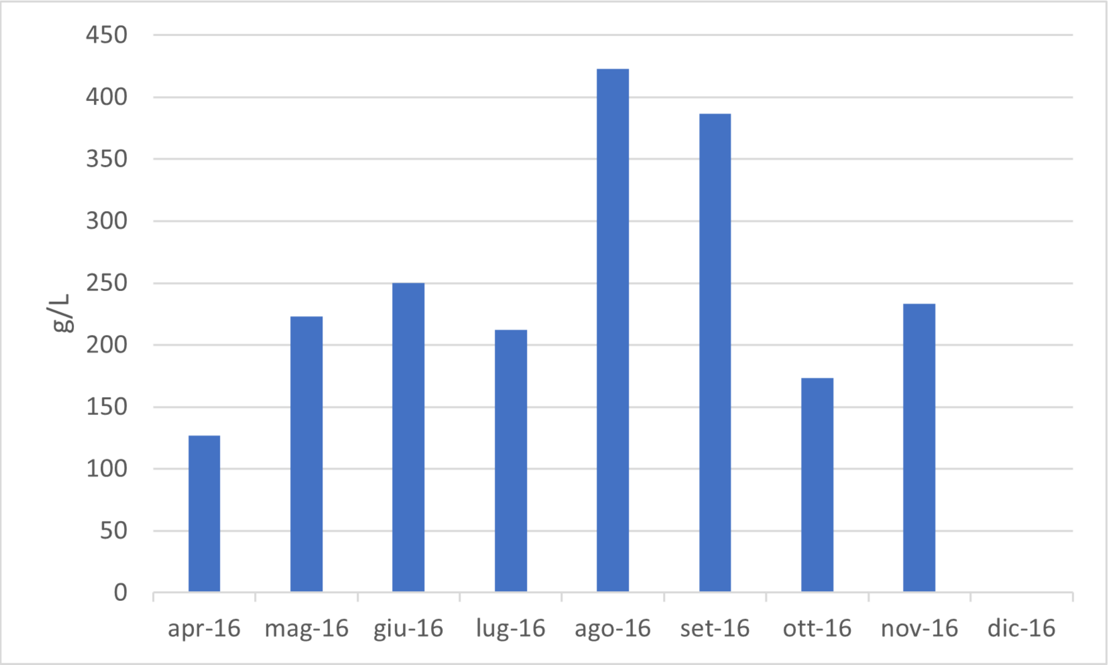

Figure SOM13: Arsenic mass released in the Popoli Gorge springs before and during the Amatrice-Norcia 2016 seismic sequences. Monthly values are expressed in g for each liter of spring discharge.
